# The Effect of Alkyl Chain Number in Sucrose Surfactant on the Physical Properties of Quercetin-Loaded Deformable Nanoliposome and Its Effect on In Vitro Human Skin Penetration

**DOI:** 10.3390/nano8080622

**Published:** 2018-08-16

**Authors:** In Ki Hong, Ji Hoon Ha, Sangkeun Han, Hakhee Kang, Soo Nam Park

**Affiliations:** 1 Department of Fine Chemistry, Cosmetic R&D Center, Seoul National University of Science and Technology, 232 Gongneung-ro, Nowon-Gu, Seoul 01811, Korea; inkiaaa@kolmar.co.kr (I.K.H.); hjh-cos@hanmail.net (J.H.H.); 2 Skin Care R&D Center, Kolmar Korea Co., Ltd., 12-11, deokgogae-gil, jeonui-myeon, Sejong 30004, Korea; sang@kolmar.co.kr (S.H.); hhkang@kolmar.co.kr (H.K.)

**Keywords:** elastic nanoliposome, edge activator, skin drug delivery system, sucrose stearate, deformability

## Abstract

Non-invasive skin penetration of a drug is increased by an edge activator, which enhances the nanoliposome deformability. The objective of this study was to investigate the role of the alkyl chain number of sucrose surfactants as an edge activator in elastic nanoliposomes. In addition, the physicochemical properties of the elastic nanoliposomes were characterized and an in vitro human skin permeation study was performed. Elastic nanoliposomes that were composed of sucrose monostearate (MELQ), sucrose distearate (DELQ), and sucrose tristearte (TELQ) were prepared using a thin-film hydration method. Particle size and entrapment efficiency of elastic nanoliposomes increased proportionally with an increase in the amounts and the numbers of the stearate in sucrose surfactant. Deformability of elastic nanoliposomes was indicated as DELQ > MELQ > TELQ and the same pattern was revealed through the in vitro human skin permeability tests. These results suggest that the number of alkyl chains of sucrose surfactant as edge activator affects the physicochemical property, stability, and skin permeability in elastic nanoliposome. Our findings give a valuable platform for the development of elastic nanoliposomes as skin drug delivery systems.

## 1. Introduction

Human skin is a very complex organ that consists of three layers; epidermis, dermis, and subcutaneous fat. The epidermis itself is composed of four sublayers, namely the basal cell layer, followed by the stratum spinosum and the granular layer, and the outermost stratum corneum (SC) layer [1]. The SC has a brick and mortar structure, comprised of multiple layers of intracellular lipid components and corneocytes (terminally differentiated keratinocytes) that play an essential role in forming the skin barrier that protects the body from external threats. However, the SC is also the biggest barrier that disturbs the absorption of ingredients through the skin. There are two routes of drug permeation through the skin, one is through skin appendages, and the other through the SC. Skin penetration route through the SC on the delivery of active ingredients is more effective than that through skin appendages, such as pores and sweat glands [2,3]. Therefore, the development of drug delivery systems, such as nanoliposome into the skin, has garnered attention.

The design of new delivery vehicles to enhance the skin barrier penetration of ingredients with therapeutic efficacy in skin diseases is a very important goal in pharmaceutical and cosmetic research. Nanoliposomes have been widely used as drug delivery systems (DDS) and have biocompatibility and biodegradability [4,5]. Recently, new delivery systems that improve the performance of nanoliposomes have been developed, such as hyalurosomes [6], glycerosomes [7], niosomes [8], and etc. However, drug delivery deep into the skin is very difficult due to the interference of the stratum corneum as skin barrier. Therefore, in order to efficiently transfer the active ingredients into the skin, development of new nanoliposome enhanced in penetration through skin barrier is required. There are typical nanoliposomes with enhanced penetration function, such as ethosome [9] and elastic nanoliposome [10].

Elastic nanoliposomes are a new skin transporter system that was proposed by G. Cevc in the early 1990s, consisting of cognitive lipids forming bilayers and surfactants, called edge activators [11]. In these structures, the surfactant acts as an edge activator to increase the radius of curvature in the elastic nanoliposome and improve elasticity and flexibility in the lipid bilayer. It has been reported that elastic nanoliposomes can penetrate across the SC and effectively transmit the active ingredient, as opposed to conventional nanoliposomes [12]. Previously, surfactants with a single carbon chain, such as cholesterol, sodium cholate, Span 80, Tween 80, and octaoxyethylene laurate ester (PEG-8-L), or ionic surfactants, such as sodium hexadecyl sulfates and cetylpyridinium chloride were commonly used [13,14,15]. These surfactants increase the membrane fluidity of the nanoliposome, exhibit high deformability properties, and increase the permeability of the skin. However, surfactants can destabilize the membrane of the nanoliposome. Recently, elastic nanoliposomes have garnered a considerable amount of attention as candidates to improve nanoliposome stability [3,16].

Recent reports describe the preparation of elastic nanoliposomes through the use of nonionic surfactants such as polyglyceryl-3-methylglucose distearate as an edge activator [17,18,19]. This surfactant has two alkyl chains, unlike an edge activator with a single alkyl chain that is used in conventional elastic nanoliposomes. Recently, various surfactants have been tested for functionality as edge activators. However, no studies have been conducted to test the nanoliposome elasticity with various numbers of alkyl chains of the edge activator.

Sucrose stearate is a non-ionic surfactant, which is used in this study, and a natural sugar-derived ingredient that has multiple functions in the cosmetics, pharmaceuticals, and food industries because it improves moisture retention and emulsion stability of nanoliposomes [20,21,22].

In this study, elastic nanoliposomes (<200 nm) were prepared with soy-derived phospholipids and sucrose surfactant acting as an edge activator, and encapsulated with the flavonol quercetin (providing antioxidative activity). More specifically, elastic nanoliposomes were prepared while using phospholipids and sucrose surfactants (sucrose monostearate, sucrose distearate, and sucrose tristearate) with different stearate ratios (100:0, 97.5:2.5, 95:5, 90:10, 80:20). The prepared elastic nanoliposomes were characterized in terms of their physiochemical properties, such as particle size, zeta potential, stability, deformability, and entrapment efficiency. In addition, morphological characteristics of the elastic nanoliposomes were determined through transmission electron microscopy (TEM). The skin permeability of the elastic nanoliposomes was evaluated while using Franz diffusion cell and confocal laser scanning microscopy (CLSM).

## 2. Materials and Methods

### 2.1. Equipment and Reagents

The soybean phosphatidylcholine (PC) (>70%) was purchased from Lipoid GmbH (Ludwigafen, Germany). Sucrose stearate was purchased from CRODA (Snaith, East Riding of Yorkshire, UK), sucrose distearate was purchased from Sigma-Aldrich (St. Louis, MO, USA) and sucrose tristearate was purchased from Stearinerie DUBOIS (Kermen, Boulgne B., France). 1,3-butylene glycol, methanol, ethanol, and chloroform of analytical grade were purchased from Sigma-Aldrich (St. Louis, MO, USA).

For the nanoliposome preparation, a rotary evaporator (Buchi, Flawil, Swizerland) and an ultrasonic shredder (Branson, MO, USA) were used. The particle size and zeta potential of the formulations were measured while using Microtrac Flex (Microtrac, North Largo, FL, USA). The deformability of nanoliposomes was measured using a mini extruder (Avanti polar lipid, Alabaster, AL, USA) and a syringe pump (KDS330 Revodix, Gyeonggi-do, Korea).

For the quantitative analysis, we used the Cary 100 ultraviolet visible (UV-vis) spectrophotometer (Vaian, Australia). For the measurement of stability of the nanoliposomes, we used the LUMiSizer 650 (LUM GmbH, Berlin, Germany). Thermal Analysis (Mettler Toledo, Columbus, OH, USA) was used for the thermal analysis. The human back skin mediated by a 52-year-old male was purchased from HansBiomed (Seoul, Korea) for the skin permeation experiment. TEM was performed while using TECNAL G Spirit Twin for morphological analysis of nanoliposomes. To evaluate the skin penetration effect of the prepared nanoliposomes, a 9 mm Franz diffusion cell (receptor volume 5 mL) and V6A Stirrer (Permegear, Bethlehem, PA, USA) were used and the confocal laser was a LSM700 from Carlp-Zeiss (Oberkochen, Germany).

### 2.2. Preparation of Elastic Nanoliposome

Elastic nanoliposomes were prepared by thin-film hydration and the composition of the nanoliposomes are shown in Table 1. Briefly, 0.002 g of quercetin was added to a round bottom flask and sucrose surfactants (mono, di, tristearate) along with phospholipids of different stearate numbers were dissolved in a mixture using 20 mL methanol/chloroform (1:1, *v*/*v*). A thick lipid membrane formed on the flask wall after the organic solvent was completely removed while using the rotary evaporator. The lipids that remained were hydrated with distilled water to form elastic nanoliposomes. To achieve a uniform particle size, the obtained elastic nanoliposomes were suspended in an ultrasonic shredder with glass for 15 min. The prepared liposomal suspension was filtered through a 0.45 μm syringe filter (Minisart CA 26 mm, Sartorius, Goettingen, Germany) to remove the unloaded quercetin in nanoliposome. The total lipid and surfactant content of the elastic nanoliposome formulation was 0.5% (*w*/*v*) and the quercetin content was 0.01% (*w*/*v*).

### 2.3. Particle Size and Zeta Potential

Particle sizes and distributions of elastic nanoliposomes were measured using Microtrac Flex, which analyzes the particle size while using light scattering. The measurement temperature was 25 °C, the scattering angle was 165°, and Argon laser was used as the light source. The mean particle size was expressed as an average value and the distribution was analyzed by using the CONTIN algorithm. The zeta potential was measured while using the same instrument.

### 2.4. Determination of Deformability of Elastic Nanoliposomes

To determine the variable deformability of the prepared elastic nanoliposome, the degree of deformability of the nanoliposome passing through an artificial permeation barrier was measured using a mini-extruder. The elastic nanoliposome was extruded at a pressure of 0.2 MPa for 1 min and the amount of the nanoliposome solution that passed through the polycarbonate membrane, with a pore size of 0.08 μm, was measured. In addition, the size of the nanoliposome particles that passed through the membrane was also measured. The elasticity of the elastic nanoliposome membrane can be described by the following Equation:

(1) $$\mathrm{Deformability}\;\mathrm{index}=J_{\mathrm{Flux}}\times {(\frac{rv}{rp})}^{2}$$

where J_Flux_ (mg/cm^2^/s) refers to the amount of nanoliposome that has passed through the membrane, *rv* refers to the particle size of the nanoliposome after extrusion, and *rp* refers to the pore size of the membrane [11,23].

### 2.5. Measurement of Entrapment Efficiency

Quercetin was removed from the elastic liposomal solution while using a 0.45 μm syringe filter (Minisart CA 26 mm). To disrupt the nanoliposome membrane, 10 mL of ethanol were added to 1 mL of the nanoliposome and the solvent was evaporated using the rotary evaporator, followed by addition of 1 mL of ethanol. Quantitative analysis of quercetin present in the elastic nanoliposomes was performed by a UV-vis spectrometry. The concentration of quercetin in elastic nanoliposomes was calculated by the calibration curve of quercetin and the absorption efficiency of the elastic nanoliposomes was calculated by the Equation below:

Loading efficiency (%) = *(T* − *P)/T* × 100 (2)

where *T* is the concentration of quercetin initially added and *P* represents the final concentration of quercetin (not filtered through a 0.45 µm syringe filter).

### 2.6. Stability of Elastic Nanoliposomes

To confirm the physicochemical stability of the manufactured elastic nanoliposome formulations, an accelerated stability test was performed while using LUMiSizer (LUM GmbH, Berlin, Germany) [24]. The samples were centrifuged at 30 °C and 1000 rpm for 5 h and measured at 865 nm wavelength at intervals of 60 s for 12 h. The instability (creaming or settling) of the sample was determined by measuring the light transmittance, and the results were expressed as an instability index. The thermal analyzer measured the thermal enthalpy (ΔH) using Thermal Analysis (Mettler Toledo, Columbus, OH, USA), starting at 30 °C and rising to 100 °C at a rate of 1 °C/min

### 2.7. Morphological Observation

Morphological characterization of the prepared elastic nanoliposomes, was performed by negative staining electron microscopy [25]. Samples were suspended onto glow-discharged copper grids. The prepared samples were adsorbed for 2 min and the buffer was removed with Whatman paper. The samples on the grids were negatively stained with 2% (*w*/*v*) uranyl acetate (UrAc) for 1 min. The sample was observed in a Technai G2 Spirit Twin microscope (FEI co., Hillsboro, OR, USA), which was operated at an accelerating voltage of 120 kV.

### 2.8. In Vitro Skin Permeation Experiment Using Franz Diffusion Cells

The skin permeation test of quercetin-coated elastic nanoliposomes was performed while using Franz diffusion cells [26]. The skin that was used in the permeation experiment was removed from the subcutaneous fat of the back of a 52-year-old male. After 5 mL of receptor phase [HCO-60:Ethanol:PBS = 2:20:78 (*w*/*v*/*v*%)] was filled in the receptor chamber, the skin was fixed between the donor and receptor phases with the stratum corneum facing up. During the experiment, the temperature was maintained at 37 ± 1 °C while using a water bath. 0.2 mL of each sample was applied to the skin surface through a donor, and 0.5 mL of each receptor phase was collected through a sampling port each time. The same amount of receptor phase was replenished in the receptor chamber. The amount of quercetin in the sample was measured with the UV-visible spectrometer. After 24 h, the skin fixed in the chamber was removed and then washed three times with PBS (Phosphate buffered saline). After washing, skin from the portion not in contact with the receptor phase was cut off and tape stripping was performed to measure the amount of quercetin remaining in the stratum corneum. The SC was removed three times by the stripping method, while using 3M Scotch tape (3M, Seoul, Korea). Ethanol (10 mL) was added to the tape and the quercetin was extracted with an ultrasonic washing machine after one hour. The ethanol was evaporated using a rotary evaporator and the extracted quercetin was dissolved in 0.5 mL of the receptor phase. After tape-stripping, the skin with the stratum corneum removed was cut with surgical scissors, and the treatment of the fine skin was performed in the same manner as the tape. The amount of quercetin in the obtained sample was measured using a UV-vis spectrophotometer.

### 2.9. In Vitro Skin Penetration Experiment (Fluorescence Image Restoration Microscopy)

To visually confirm the skin permeation efficiency of the elastic nanoliposome, a fluorescence image restoration microscope was used. The elastic nanoliposomes were prepared by a thin-film hydration method, as described above, and 0.015% of rhodamine B and FITC (fluorescein isothiocyanate) were simultaneously collected. Rhodamine B and FITC, which were not loaded in the elastic nanoliposome, were removed while using a 0.45 μm syringe filter. Human skin with stratum corneum and dermal layer was used for the permeation experiment. Preparation of Franz diffusion cells was carried out, as described above. 0.5 mL of each sample was added to the skin through the donor, and after 12 h, the skin was taken out and washed three times with PBS. After washing, the portion of skin not in contact with the receptor phase was cut out, and the tissue was frozen at −70 °C by treating the cryo mold with an Optical coherence tomography (OCT) embedding matrix (CellPatch Ltd., Powys, Wales, UK).

The frozen tissue was cross-sectioned at a thickness of 10 μm at −20 °C while using a cryotome (LEICA CM1860, Wetzlar, Germany). To stain the nuclei of skin cells, 10 μg/mL of 4′,6-diamidino-2-phenylindole (DAPI) was added to the fixed tissue slides and incubated for 20 min at room temperature. Polyvinyl alcohol mooring medium with BABCO (Sigma) was applied to reduce the photobleaching phenomenon and increase the adhesion between skin tissue and cover glass.

Delta Vision RT (Applied Precision, Issaquah, WA, USA) was used to determine the degree of penetration of the fluorescent reagent, and skin tissue was observed with an Olympus IX70 Inverted Microscope. The wavelengths for Rhodamine B and FITC were 490/20 and 430/10 nm, and the emission wavelengths were 528/38 and 470/30 nm, respectively. The excitation and emission wavelengths for DAPI were 360/40 and 457/50 nm, respectively.

## 3. Results and Discussion

### 3.1. Particle Size and Zeta Potential

Elastic nanoliposomes with varying ratios of PC and sucrose surfactant (100:0, 95:5, 90:10, 85:15 and 80:20) were prepared, and conventional nanoliposome (CLQ) was prepared with 100% PC and quercetin (Table 1). Elastic nanoliposomes were prepared according to the number of stearates of sucrose surfactant and quercetin; sucrose monostearate (MELQ1–4), sucrose distearate (DELQ1–4), and sucrose tristearate (TELQ1–4). Table 2 shows the particle size, polydispersity index (PDI) and zeta potential of the nanoliposomes.

In terms of particle size, elastic nanoliposomes (MELQ, DELQ, and TELQ) were larger than CLQ. Elastic nanoliposomes increased in average particle size as the content of sucrose stearates derivatives increased from 2.5% to 10% (*w*/*w*). Elastic nanoliposomes also increased in particle size as the number of stearates of sucrose surfactant increased (TELQ > DELQ > MELQ). However, the particle size of elastic nanoliposomes decreased when the content of sucrose surfactant was 20%. It is plausible that the excess of sucrose surfactant caused the collapse and solubilization of the lipid membrane composed of PC. The PDI of elastic nanoliposomes was monodispersed (<0.3).

The zeta potential represents the charge on the particle surface of the nanoliposome and affects the stability. An increase in the zeta potential of nanoliposome enhances repulsive force between nanoliposomes, which interferes with the aggregation of particles [27]. CLQ indicated a zeta potential value of −13 mV, and the value of MELQ1–4 (−14.2~−12.4 mV), DELA1–4 (−12.0~−10.3 mV), and TELQ1–4 (−11.0~−10.1 mV). As the number of stearates of sucrose surfactant increased or the content increased from 2.5% to 10%, the zeta potential of elastic nanoliposome decreased slightly.

### 3.2. Stability Evaluation

**Figure 1:**
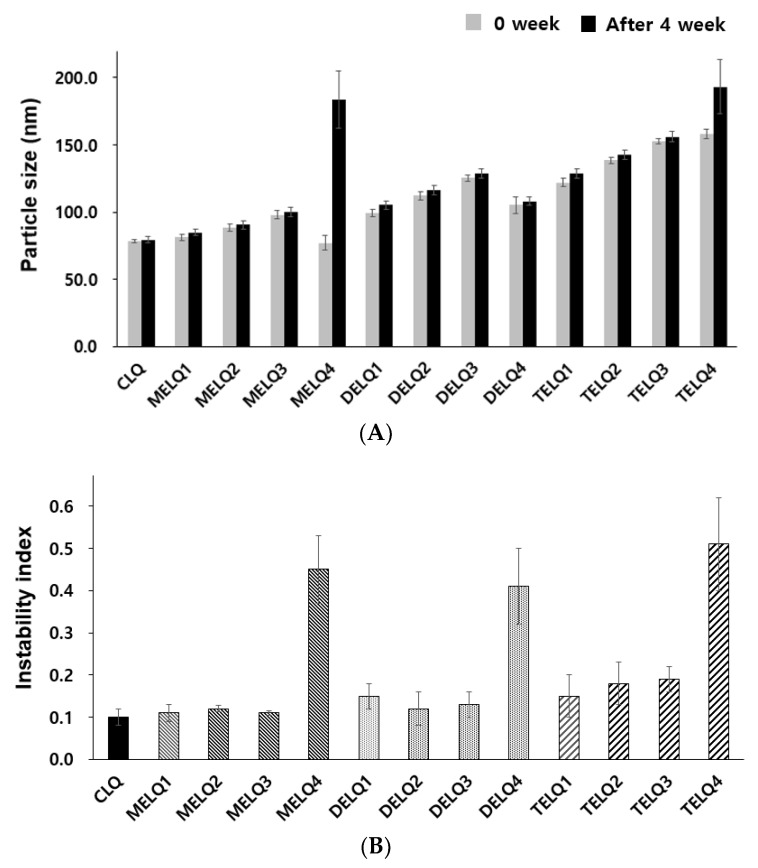
Stability of nanoliposomes. (**A**) Particle size for nanoliposomes after 0 week and 4 weeks. (**B**) Instability index.

The change of particle size in relation to the storage period is an important factor to evaluate the stability of the nanoliposome [28]. In general, unstable nanoliposomes tend to agglomerate with each other and tend to increase in particle size. To evaluate the storage stability of the nanoliposomes, particle sizes were compared immediately after the nanoliposomes were prepared and after storage for four weeks at 4 °C (Figure 1A).

CLQ and DELQ1–4 showed no change in particle size after storage for four weeks at 25 °C. MELQ and TELQ with a sucrose surfactant of less than 10% were stable for four weeks, but MELQ4 and TELQ4 with a surfactant content of 20% were unstable.

We further performed an accelerated stability test using a LUMiSizer to evaluate the stability of the nanoliposomes. This instrument uses space and time-resolved extinction profiles (STEP) techniques while using centrifugation to quickly form non-mixed environments of dispersed systems [29]. The instability (creaming or sedimentation) of a nanoliposome was determined by measuring the light transmittance, and the results were expressed as an instability index. The instability index is a value between 0 and 1, 0 is stable above the level of light transmission, and 1 represents complete phase separation, thus exhibiting the lowest stability in centrifugal force [30]. We measured the instability index of the nanoliposomes by stressing them at 30 °C to 1000 RPM for 12 h and measured the transmittance at 60 s intervals.

The instability index of CLQ was 0.1, which indicates a very stable form. Elastic nanoliposomes containing 2.5–10% of sucrose surfactants were stable, but the instability index of elastic nanoliposomes containing 20% (MELQ4, DELQ4, and TELQ4) was determined to be over 0.4, indicating a very unstable structure. In particular, TELQ showed higher levels of instabillity overall than other elastic nanoliposomes. DELQ4 was stable for one month in the storage stability evaluation, but it was unstable in the acceleration stability evaluation. These results suggest that the surfactant concentration range that is required for the stable preparation of sucrose surfactant-based elastic nanoliposomes is within 10%.

### 3.3. Drug Entrapment Efficiency

The entrapment efficiency of quercetin encapsulated in nanoliposomes is shown in Table 2. The entrapment efficiency of CLQ was 61.2%. The efficiency of quercetin absorption of elastic nanoliposomes was higher than that of CLQ. As the number and content of stearates of sucrose surfactants composing elastic nanoliposomes increased, quercetin uptake efficiency also increased. The quercetin entrapment efficiency of elastic nanoliposomes was proportional to the particle size. However, the entrapment efficiencies of MELQ4 and DELQ4 with 20% sucrose surfactants were 62.5% and 80.8%, which were lower than that of MELQ3 (78.7%) and DELQ3 (84.2%) containing 15% surfactant. It is regarded that the absorption efficiency of the elastic nanoliposome is relatively decreased by dispersing the quercetin in the solvent by the micelle that is formed due to the excessive amount of the surfactant [31].

### 3.4. Deformability

In the process of passing through the lipid layer between the corneocytes of the SC, the nanoliposome experiences physical stress due to space limitations. The greater the deformability of the nanoliposome, the more flexible it is. Increased flexibility allows for nanoliposomes to adapt to these stresses and pass easily through the stratum corneum of the skin, resulting in deeper delivery of the active ingredients into the skin [32]. The extrusion method [33] was used to evaluate the deformability of the nanoliposomes, and the deformability index was determined while using Equation (1) (Table 1).

The deformability index of CLQ was 5.2 and the deformability indices of elastic nanoliposomes were three times higher than CLQ. In particular, DELQ showed the highest deformability index, followed by MELQ and TELQ. This value was also increased as the content of sucrose surfactant in elastic nanoliposomes increased from 2.5% to 10% and was proportional to the particle size of the nanoliposome. However, MELQ4 and DELQ4, containing 20% sucrose surfactant, had a smaller particle size than MELQ3 and DLQ3, but it showed no significant difference in deformability. In the evaluation of stability against physical stress, MELQ4 and DELQ4, which showed high levels of stress, were easily disrupted during extrusion, resulting in large J_Flux_ value and large deformability index. Based on these results, elastic nanoliposomes MELQ3, DELQ3, and TELQ3 containing 10% of sucrose surfactant with high deformability and storage stability, including high entrapment efficiency, were selected to test their stability.

**Figure 2:**
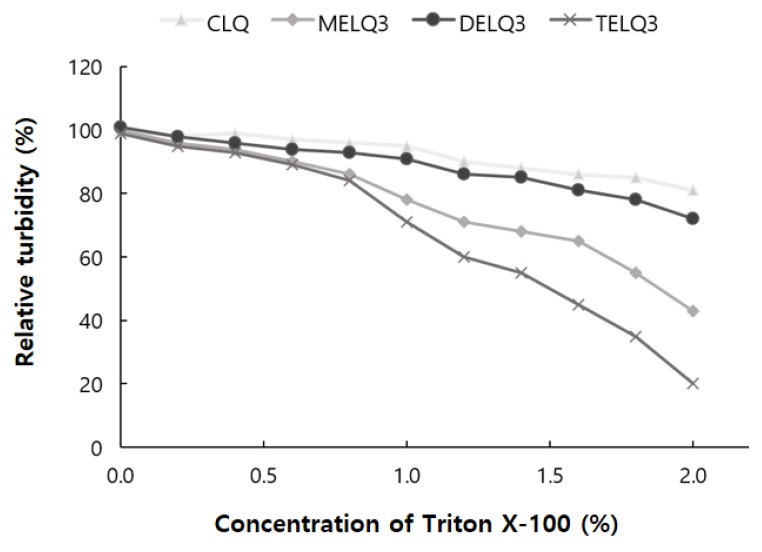
Relative turbidity of nanoliposomes (CLQ, MELQ3, DELQ3, and TELQ3) at different concentrations of Triton X-100.

To evaluate the stability of the final selected elastic nanoliposomes to surfactant stress, Triton X-100 was used. Triton X-100 is a non-ionic surfactant that is composed of hydrophilic groups of polyethylene oxide and hydrophobic groups of aromatic hydrocarbons. Triton X-100 solubilizes the lipids of nanoliposome vesicles and makes them transparent [34]. The turbidity of the nanoliposome solution was measured at a wavelength of 500 nm with a UV-vis spectrophotometer. To evaluate the stability of elastic nanoliposomes, they were treated with Triton X-100 concentrations between 0 and 2% and turbidity changes were observed (Figure 2).

As the treatment concentration of Triton X-100 increased from 0 to 2%, the turbidity of CLQ decreased from 100% to 81%. The turbidity of elastic nanoliposomes that were treated with Triton X-100 was lower than that of CLQ. At 2% Triton X-100, the turbidity of MELQ3 and TELQ3 was reduced to 43% and 20%, respectively. However, DELQ3 was found to be stable with relatively higher turbidity than MELQ3 and TELQ3. These results propose that the stability of elastic nanoliposomes is in order of DELQ3 > MELQ3 > TELQ3.

### 3.5. Crystallinity of Nanoliposomes

**Figure 3:**
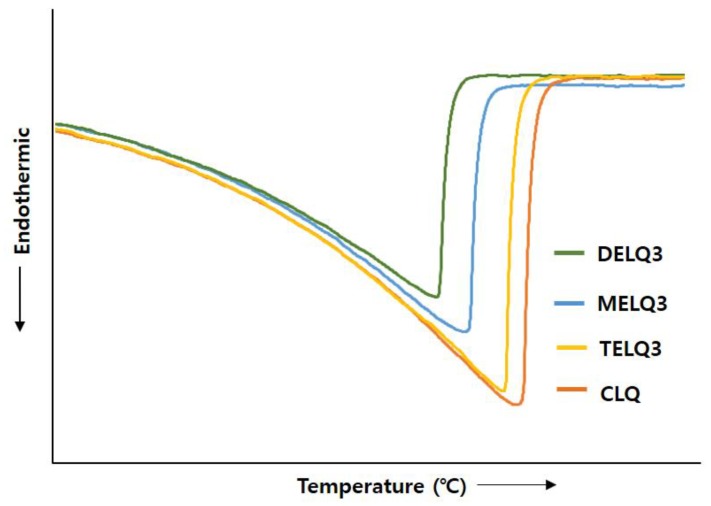
Effect of nanoliposomes on the differential scanning calorimetry (DSC) thermograms of conventional nanoliposome (CLQ), MELQ3, DELQ3, and TELQ3.

Surfactants that were added to nanoliposomes interfere with the association between lipids in nanoliposomes, resulting in a decrease in crystallinity [35]. Reduction of crystallization increases the deformability of the nanoliposome, which in turn increases its fluidity [32]. Therefore, to evaluate the crystallinity of nanoliposomes the transition enthalpy (ΔH°) and the peak temperature (T_peak_) between normal nanoliposome (CLQ) and elastic nanoliposomes were compared while using differential scanning calorimetry (DSC) (Figure 3).

The ΔH° of CLQ was 2173.32 J/g and T_peak_ was 73.26 °C. DELQ3 showed a slightly lower transition enthalpy change (ΔH° = 85.38 J/g), and a peak temperature change (ΔT_peak_ = 1.11 °C) higher than CLQ. In contrast, the transition enthalpy changes of MELQ3 and TELQ3 when compared to CLQ were 400.4 and 844.23 J/g and the ΔT_peak_ were 3.49 and 6.04 °C, respectively. The differences in the transition enthalpy and peak temperature of CLQ and elastic nanoliposomes were in the order of TELQ3 > MELQ3 > DELQ3. These results were in contrast to the stability tendency of elastic nanoliposomes, and the variability was proportional. These results suggest that the decrease of the melting point of elastic nanoliposomes by sucrose surfactant weakened the crystallinity and increased the membrane fluidity of the nanoliposome, increasing the deformability of elastic nanoliposomes.

### 3.6. Morphological Observation of Nanoliposomes

**Figure 4:**
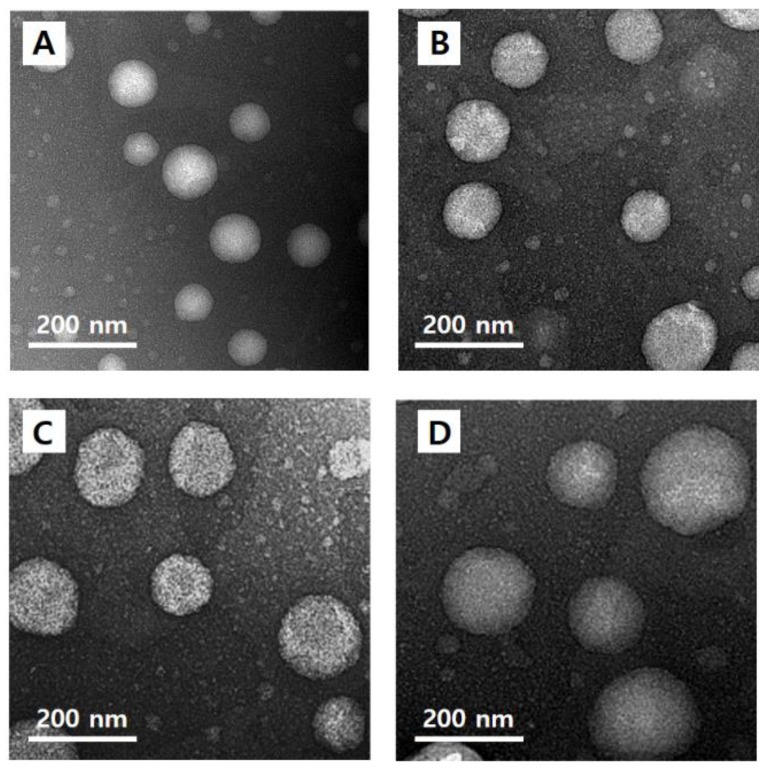
Transmission electron microscopy (TEM) images of (**A**) CLQ, (**B**) MELQ3, (**C**) DELQ3, and (**D**) TELQ3.

The morphology of quercetin-loaded nanoliposomes (CLQ, Figure 4A) and elastic nanoliposomes MELQ3 (Figure 4B), DELQ3 (Figure 4C), and TELQ3 (Figure 4D). The shape of the nanoliposomes was regular spherical, and the particle size was similar to the average value that was measured by particle size analyzer.

### 3.7. In Vitro Skin Permeation Test

To evaluate the skin absorption of elastic nanoliposomes (MELQ3, DELQ3, and TELQ3) containing 10% sucrose surfactant, Franz diffusion cells were used. Quercetin-loaded 1,3-BG, CLQ, and elastic nanoliposomes were applied to human skin, and the average penetration rate (%) of skin permeation amount and skin deposition of quercetin according to treatment time was evaluated. 1,3-BG was used as a control solution and CLQ was used as a control nanoliposome.

**Figure 5:**
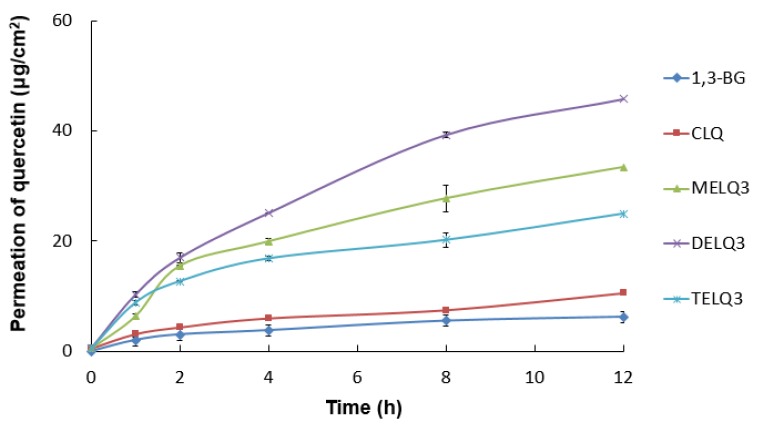
In vitro skin permeation profiles of 1,3-butylene glycol solution (1,3-BG), conventional nanoliposome (CLQ) and elastic nanoliposomes (MELQ3, DELQ3, and TELQ3) though Human skin for 12 h.

The permeation amount of quercetin over time (0~12 h) within a specific area (0.6362 cm^2^ is shown in Figure 5. Briefly, elastic nanoliposomes showed higher skin permeability than CLQ and the skin permeability of elastic nanoliposomes was in the order of DELQ3 > MELQ3 > TELQ3. In particular, the amount of quercetin permeating the skin in DELQ3 increased significantly with time. These results were consistent with the deformability index results of elastic nanoliposomes (Table 1).

**Figure 6:**
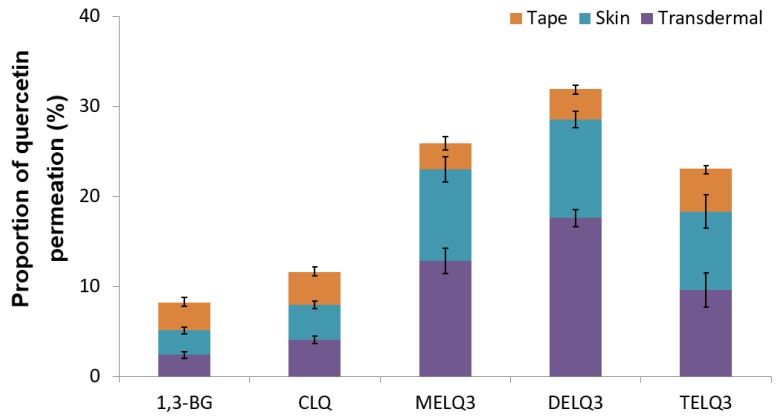
Proportions of permeated amount (%) of quercetin in 1,3-butylene glycol solution (1,3-BG), conventional nanoliposome (CLQ) and elastic nanoliposomes (MELQ3, DELQ3, and TELQ3) after 12 h. (Tape: stratum corneum, Skin: epidermis without stratum corneum and dermis, Transdermal: permeated through skin).

Figure 6 shows the permeated amount of quercetin over a 12-hour period. The amount of quercetin in the skin (epidermis and dermis), except the stratum corneum (Transdermal). The amount of quercetin contained in elastic nanoliposomes was higher than that of CLQ and 1,3-BG. In particular, they carried relatively greater amounts of quercetin in the skin. The amount of quercetin that was encapsulated into the prepared elastic nanoliposomes was DELQ3 > MELQ3 > TELQ3, and this was reflected which showed the same tendency as the skin permeability. The high accumulation of quercetin in the skin is highly advantageous because it can provide the concentration that is needed to demonstrate the efficacy of quercetin. TELQ3 transported a small amount of quercetin into the skin both on the skin and transdermal side, while the stratum corneum absorbed a large amount. These results suggest that the interaction between TELQ3 and the stratum corneum enhanced the adsorption of quercetin by the sucrose tristearate.

### 3.8. In Vitro Skin Penetration Experiment (Fluorescence Image Restoration Microscopy)

**Figure 7:**
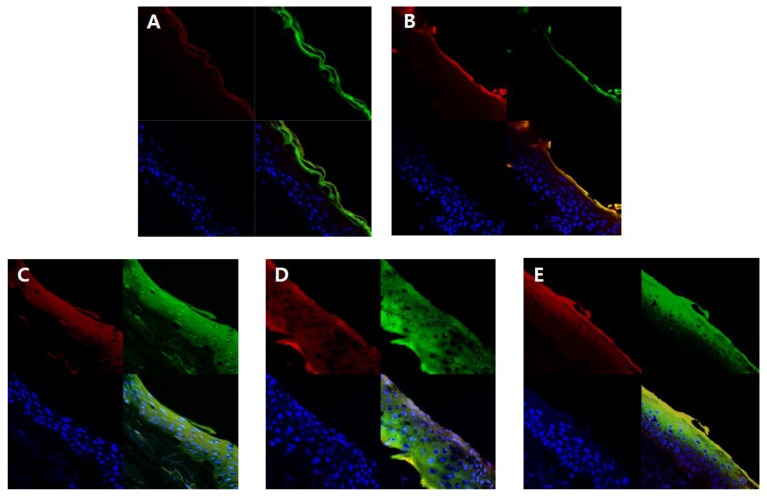
Fluorescence images of a cross-section of human skin incubating with nanoliposomes containing Rhodamine B (red) and fluorescein isothiocyanate (FITC) (green) for 12 h. Cellular nucleuses were stained with 4′,6-diamidino-2-phenylindole (DAPI) (blue). (**A**) 1,3-butylene glycol solution, (**B**) conventional nanoliposome, CLQ, (**C**) elastic nanoliposome (Sucrose stearate), MELQ3, (**D**) elastic nanoliposome (Sucrose distearate), DELQ3, (**E**) elastic nanoliposome (Sucrose tristearate), TELQ3.

In order to visually confirm the skin permeability of elastic nanoliposomes (MELQ3, DELQ3, and TELQ3), rhodamine B (red), which is a water soluble fluorescent substance, and FITC (green), which is a fat soluble fluorescent substance, the substances observed with confocal laser scanning microscopy (CLSM) (Figure 7). CLQ, which is a conventional nanoliposome, was used as a control.

The amount of lipid soluble FITC attached to 1,3-BG and CLQ was found to be abundant in the stratum corneum. However, it did not penetrate deeper into the epidermis and dermis (Figure 7A,B). CLQ delivered water soluble rhodamine B to the stratum corneum and epidermis, but 1,3-BG hardly penetrated the skin. This suggests that CLQ can adsorb or transfer water-soluble and lipid-soluble components to the skin due to the biocompatibility of nanoliposomes, but 1,3-BG does not significantly affect skin absorption.

In contrast, elastic nanoliposomes delivered rhodamin B and FITC to the dermal layer (Figure 7C–E). The order of depth of fluorescent materials loaded on elastic nanoliposomes was DELQ3 > MELQ3 > TELQ3. Specifically, DELQ3 delivered rhodamin B and FITC throughout the skin. MELQ3 and TELQ3 delivered fluorescent substances from the stratum corneum to the upper dermis. Furthermore, TELQ3 adsorbed a large amount of fluorescent substances on the stratum corneum. This was because sucrose tristearate, which is an edge activator of TELQ3, increased deposition on the skin by increasing fusion of nanoliposome membrane and stratum corneum [36]. These results show that an elastic nanoliposome (DELQ3) containing sucrose distearate, which shows excellent deformability, entrapment efficiency, and stability, is very useful as a delivery vehicle for the skin transfer of quercetin.

In conclusion, highly elastic nanoliposomes with variable shape are more useful for delivering hydrophilic substance to skin than general nanoliposomes. In addition, the number of stearates of sucrose surfactants acting as edge activators in elastic nanoliposomes has a diverse effect on the drug delivery to skin.

## 4. Conclusions

In this study, sucrose stearate derivatives (sucrose monostearate, distearate, tristearate) were used in quercetin-loaded elastic nanoliposomes to evaluate the effect of alkyl chain number of the edge activator on the physiochemical properties and skin permeation of prepared nanoliposomes. The results of our study suggest that the characteristics of elastic nanoliposomes differ according to the number of alkyl chains of the sucrose surfactant. These results provide important information that could be used to develop a novel edge activator for elastic nanoliposomes. Especially, DELQ3, composed of 10% sucrose distearate and PC, possessed the lowest crystallization and highest deformability and delivery of quercetin into skin of all nanoliposomes. Thus, DELQ3 is an optimized drug delivery system for an active ingredient, such as quercetin.

## Figures and Tables

**Table 1:** Composition of various nanoliposomes containing quercetin.

Formulation Code	PC ^a^ (*w*/*w*%)	Sm ^b^ (*w*/*w*%)	Sd ^c^ (*w*/*w*%)	St ^d^ (*w*/*w*%)	Quercetin (*w*/*w*%)
CLQ	100.0	-	-	-	0.002
MELQ1	97.5	2.5	-	-	0.002
MELQ2	95.0	5.0	-	-	0.002
MELQ3	90.0	10.0	-	-	0.002
MELQ4	80.0	20.0	-	-	0.002
DELQ1	97.5	-	2.5	-	0.002
DELQ2	95.0	-	5.0	-	0.002
DELQ3	90.0	-	10.0	-	0.002
DELQ4	80.0	-	20.0	-	0.002
TELQ1	97.5	-	-	2.5	0.002
TELQ2	95.0	-	-	5.0	0.002
TELQ3	90.0	-	-	10.0	0.002
TELQ4	80.0	-	-	20.0	0.002

^a^ Soy phosphatidylcholine, ^b^ Sucrose monostearate, ^c^ Sucrose distearate, ^d^ Sucrose tristearate.

**Table 2:** Mean Particle Size, Polydispersity Index, Zeta Potential, Encapsulation Efficiency, and Deformability Index of Various Nanoliposomes.

Formulation Code	Size(nm)	Polydispersity Index	Zeta Potential (mV)	Encapsulation Efficiency (%)	Deformability Index
CLQ	78.4 ± 1.2	0.182 ± 0.005	−13.1 ± 0.1	61.2 ± 2.7	5.2 ± 0.5
MELQ1	81.2 ± 2.2	0.212 ± 0.005	−14.2 ± 0.4	69.3 ± 1.2	18.4 ± 2.4
MELQ2	88.3 ± 2.7	0.218 ± 0.007	−13.5 ± 0.3	75.3 ± 2.3	20.2 ± 3.3
MELQ3	98.0 ± 3.0	0.242 ± 0.004	−13.2 ± 0.4	78.7 ± 3.2	23.3 ± 1.7
MELQ4	77.3 ± 5.5	0.266 ± 0.011	−12.4 ± 1.5	62.5 ± 2.9	17.5 ± 3.2
DELQ1	99.3 ± 2.6	0.178 ± 0.003	−12.0 ± 0.5	76.3 ± 1.2	18.6 ± 1.2
DELQ2	112.2 ± 3.3	0.188 ± 0.004	−11.5 ± 0.4	81.2 ± 2.3	21.7 ± 3.2
DELQ3	125.3 ± 2.6	0.189 ± 0.004	−11.2 ± 0.9	84.2 ± 3.2	25.5 ± 1.2
DELQ4	105.2 ± 6.2	0.212 ± 0.016	−10.3 ± 1.2	80.8 ± 3.1	17.6 ± 1.0
TELQ1	122.0 ± 3.1	0.231 ± 0.007	−11.0 ± 0.3	77.4 ± 1.2	14.5 ± 1.1
TELQ2	138.6 ± 2.5	0.264 ± 0.006	−10.9 ± 0.3	83.6 ± 2.3	16.5 ± 2.1
TELQ3	152.7 ± 2.1	0.282 ± 0.009	−10.5 ± 0.7	86.5 ± 3.2	19.6 ± 1.2
TELQ4	158.2 ± 3.6	0.278 ± 0.021	−10.1 ± 1.8	89.5 ± 2.2	21.2 ± 3.2
